# Long-range polymer ordering by directional coating to remarkably enhance the charge carrier mobility in PCDTPT-based organic field-effect transistors

**DOI:** 10.1098/rsos.240153

**Published:** 2024-05-22

**Authors:** M. Javaid Iqbal, Kashif Saghir, Tahmina Afzal, Badriah S. Almutairi, M. Zahir Iqbal, Mohsin Ali Raza, Saira Riaz

**Affiliations:** ^1^ Centre of Excellence in Solid State Physics, University of the Punjab, Lahore 54590, Pakistan; ^2^ Department of Physics, College of Science, Princess Nourah bint Abdulrahman University, P.O.Box 84428, Riyadh 11671, Saudi Arabia; ^3^ Faculty of Engineering Sciences, GIK Institute of Engineering Sciences and Technology, Topi, Khyber Pakhtunkhwa 23640, Pakistan; ^4^ Institute of Metallurgy and Materials Engineering, University of the Punjab, Lahore 54590, Pakistan

**Keywords:** organic field-effect transistors, molecular assembly, brush coating, nanogrooves patterning, directional coating, polymer alignment

## Abstract

With the wide potential of organic field-effect transistors in all the modern electronic circuitries, researchers are grappling with the challenge of poor charge transport and hence lower mobility in organic polymers. Low-charge carrier mobility is mainly due to disorder in the molecular packing of organic semiconductors along with other factors, such as impurities, defects and interactions between molecules. The current research work has been conducted to align the molecular chains of poly[4-(4,4-dihexadecyl-4H-cyclopenta[1,2-‌‌‌b:5,4-‌b′]‌dithiophen-2-yl)-alt-[1,2,5]thiadiazolo-[3,4-c]pyridine] (PCDTPT) using directional coating techniques such as dip coating and brush coating on nano-grooved substrates. Long-range order of polymer chains was clearly observed along the direction of brush coating and nanogrooves in optical and atomic force microscope (AFM) images while transmission spectra confirmed decreased pi–pi stacking for the polymer films deposited by this technique. By comparing the mobility performance of brush-coated devices with other techniques, we found a remarkable mobility enhancement of 90 times that of conventional spin-coated device and 24 times enhancement compared with the dip-coated device for the case when the alignment of polymer chains was parallel to the channel. All the fabrication and characterizations were performed in the ambient environment. This study demonstrates a potential approach to align the polymers on long and short ranges hence providing a route for high-performing devices in ambient conditions.

## 1. Introduction

In the past three decades, organic field-effect transistors (OFETs) have embarked upon the immense interest of researchers owing to their solution processibility, low-temperature processing, low-cost fabrication and compatibility with plastic substrates [1–6]. The research efforts have resulted in significant breakthroughs in the performance of OFETs based on π-conjugated organic semiconductors (OSCs) including small molecules and polymers. However, there are some research challenges that need to be addressed before large-scale commercialization is possible. One of the hindrances in the realization of high-performance OFETs is the intrinsic low-charge carrier mobility of OSCs material. Mobility has improved over two decades from 10^−5^ to 10 cm^2^ V^−1^ s^−1^, which is 10 times more than that for FETs based on amorphous silicon thin film transistor technology (0.5–1 cm^2^ V^−1^ s^−1^) [7–11]. Such substantial enhancements in the performance of OFETs have enabled inspiring low-cost applications of flexible electronics like organic light emitting diodes, e-paper displays, chemical and biological sensors and RF identification tags. Among pi-conjugated polymers, donor–acceptor (D-A) copolymers are active in research owing to their high charge transport properties due to tight pi–pi stacking of alternate chains [3,12–15]. These polymers are composed of donor and acceptor moieties where the strong interaction between the donor unit of one molecule and with acceptor unit of other molecule results in reduced intermolecular distance [16,17].

It has been reported that fabrication methods considerably affect the alignment and mobility in conjugated polymers wherein the polymer film is generally deposited through the solution processing method rather than the vacuum deposition [18–20]. Fabrication of solution-processed polymer FETs is a low-cost solution for applications like flexible displays, healthcare and large-area electronics but this method increases the level of polymer disorder. Furthermore, device mobility strongly depends upon solvent, solution concentration, deposition temperature, post-deposition annealing temperature and deposition method [16,21–23]. All these factors affect the molecular packing of deposited material. The alignment in molecular packing results in a reduction in pi–pi stacking distance and hence an increase in charge transport [24–26]. So, for long-range alignment of the molecular backbone and side chains, controlled deposition techniques are required and nano-patterning of the substrate prior to polymer deposition is a potential directional deposition technique among them [27,28]. Lee *et al*. [29] have achieved mobility value as high as 50 cm^2^ V^−1^ s^−1^ by employing nano-grooved patterning on SiO_2_ dielectric and controlled deposition of D-A polymer poly[4‐(4,4‐dihexadecyl‐4H‐cyclopenta[1,2‐b:5,4‐b′]dithiophen‐2‐yl)‐alt‐[1,2,5]thiadiazolo‐[3,4‐c]pyridine] (PCDTPT) using sandwich casting system in controlled environment in which PCDTPT vapours reached the substrate (hydrophobic substrate) by tunnelling through glass separator through capillary action. They commented that the slow deposition of polymer using this process resulted in long chain ordering along the nanogrooves. This method is slow and not suited for large-area fabrication of organic devices. Bulgarevich *et al*. [27] used spin coating method for the deposition of poly(2,5‐bis(3‐hexadecylthiophene‐2‐yl)thieno[3,2‐b]thiophene) (pBTTT‐C16) on hydrophobic Si/SiO_2_ nano-grooved substrate. As the spin coating is an isotropic technique, to restrict the spreading of the solution near edges as they claimed that near edges pi–pi stacking was aligned with gate dielectric/active layer interface and increased the charge transport, they restricted the edges of substrate as hydrophilic. They obtained the mobility increase by factor of 2 as compared with isotropic spin-coated devices. Spin coating is also not suited for large-area electronics and it has its own limitations depending upon the substrate treatment and solution spreading criteria during coating.

For low-cost and directional deposition of organic materials through solution processing for large-area electronics, the use of a paintbrush for polymer deposition was introduced [30,31]. Lin *et al*. used a Chinese brush to paint the Poly[2,5-(2-octyldodecyl)-3,6-diketopyrrolopyrrole-alt-5,5-(2,5-di(thien-2-yl)thieno [3,2-b]thiophene)], (DPPDTT) polymer on a Si/SiO_2_ nano-grooved hydrophobic substrate. They had shown a six times increase in mobility value as compared with spin-coated devices on nano-grooved substrate. They also showed that brush coating along the parallel channel device had higher mobility as compared with the perpendicular channel device [31].

In this research, we took advantage of above-mentioned low-cost brush coating technique on the nano-grooved substrate for obtaining long-range ordering of molecular chains using Si/SiN substrate and PCDTPT as polymer which had shown promising results on nano-grooved substrate and deposition using sandwich casting system. Brush-coated PCDTPT polymer chains were aligned along the direction of the brush which increased charge transport. We used a shadow mask for contact deposition which was patterned such that four devices were deposited on a single substrate; two devices had a channel perpendicular to the other two. We observed that the devices that have polymer chains aligned parallel to the channel length have higher mobility as compared with perpendicular alignment. We also fabricated devices using conventional spin coating and dip coating techniques for detailed comparison with brush coating on nano-grooved substrates. The devices based on brush coating on the nano-grooved substrate have shown 90 times higher mobility compared with the conventional spin method. Dip coating also showed two times improvement in mobility but the enhancement is much lower than brush coating. Furthermore, for the chain alignment parallel to the channel, the increase in mobility is higher by dip coating and brush coating methods. All the fabrication and characterizations were performed in the ambient environment. Our study suggested the practical realization of the brush coating technique in ambient conditions for large-area applications.

## 2. Experimental details

**Figure 1.**
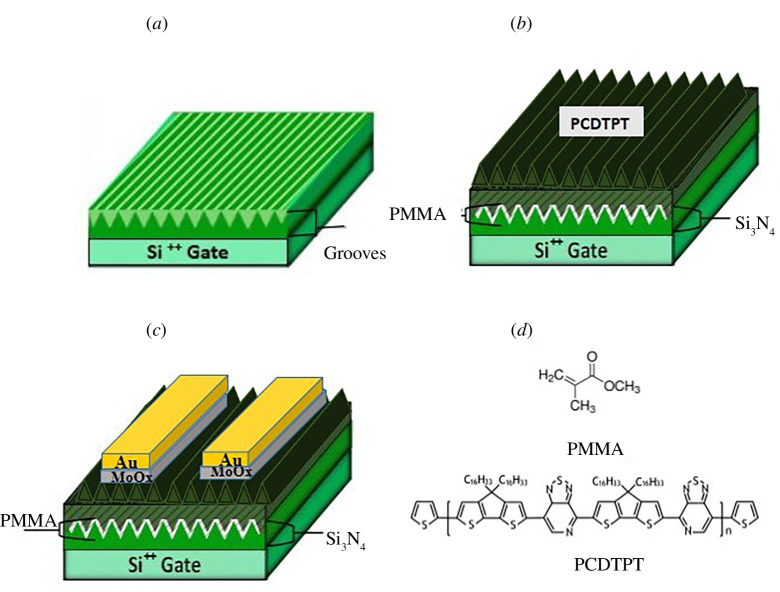
Schematic representations (*a*) grooves on the substrate. (*b*) PCDTPD on grooved substrate. (*c*) Complete device on grooved substrate. (*d*) Chemical structure of polymethyl methacrylate (PMMA) and PCDTPT, respectively.

We used highly doped <100> n-Si wafers with dimensions of 15 × 15 mm with approximately 400 nm thick Si_3_N_4_ layer as the gate dielectric. Nanogrooves were prepared on a gate dielectric insulator (Si_3_N_4_) by rubbing the diamond lapping film having a particle height of 5 nm. The substrates were cleaned to remove any residual contamination by ultrasonication for 15 min each in acetone and isopropyl alcohol (IPA). The cleaned substrates were blown dry with pressurized nitrogen gas. The solutions of organic material PCDTPT and polymethyl methacrylate (PMMA) were prepared in chlorobenzene (CB) (13 mg ml^−1^) and toluene (5 mg ml^−1^), respectively. In order to completely dissolve PCDTPT and PMMA, solutions were magnetically stirred at 50°C for 12 h. PMMA was deposited by spin coating the PMMA solution at 1000 r.p.m. for 60 s on nano-grooved substrates. Then samples were annealed in a furnace at 150°C for 15 min. The thickness of the deposited PMMA dielectric layer is about 35 nm measured by using a surface profilometer after the baking of the PMMA layer. PCDTPT solution was brush coated on nano-grooved substrates with a brushing speed of 8 mm s^−1^ while substrate temperature was kept to be 50°C (by placing the substrate on a hot plate during the coating process). The samples were dry annealed at a temperature of 100°C for 20 min in a nitrogen environment after coating. Figure 1*a*,*b* shows schematics of grooves and subsequently coated layers. For spin coating devices, PCDTPT solution was spin-coated (1000 r.p.m. for 60 s) on plain substrates and dry annealed at 100°C for 20 min in a nitrogen environment. For dip coating devices, cleaned nano-grooved substrates were vertically dipped in a PCDTPT solution bath with a dip withdrawal speed of 5 mm s^−1^ and immediately dry annealed at 100°C for 20 min in a nitrogen environment. Gold contacts with molybdenum oxide (MoO_2_) Au/MoO_x_ for adhesion of gold contacts were deposited through a shadow mask in a thermal evaporator chamber at a vacuum of 10^−6^ mbar. All handling was done in an ambient environment unless otherwise specified. Structural characterization was performed with an optical microscope and tapping mode atomic force microscope (AFM). Electrical measurements were performed using a Keithley 4200SCS parameter analyser with four probe stations. Figure 1*c* shows a schematic of a fully fabricated device on the grooved substrate (other devices share a similar configuration) and figure 1*d* shows the molecular structures of PCDTPT and PMMA.

## 3. Results and discussion

### 3.1. Structural characterization

**Figure 2.**
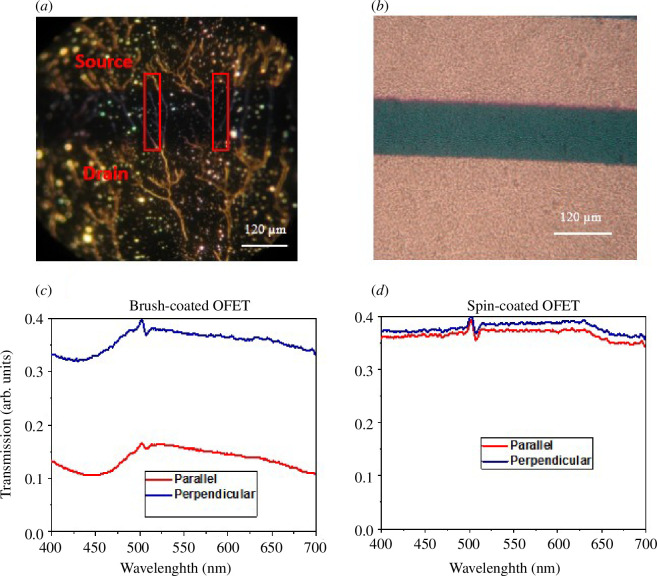
(*a*) Optical microscope image of brush-coated OFET on plain substrate. Long-range order of molecular packing can be clearly seen from image. Most of the long chains are continuous through the channel. (*b*) Optical microscope image of spin-coated OFET, no chain alignment of PCDTPT can be observed from image (shining area showed the gold contacts). (*c*) Transmission spectra of brush-coated device when polymer chains orientation was parallel and perpendicular to incident light. (*d*) Transmission spectra of spin-coated device.

The morphological properties and microstructure of the resulted PCDTPT brush-coated thin films were first studied using an optical microscope. Figure 2*a* shows the microscopic alignment of chains on plain Si substrate, that is along the source-to-drain contacts. Some of these chains covered the whole channel length and drain–source contacts as shown in the figure. In order to see the difference, the morphology of spin-coated PCDTPT thin film was also examined with an optical microscope. The microscopic images of spin-coated PCDTPT thin film exhibited granular morphology without any long-range features. Figure 2*b* shows the gold regions in optical microscope images due to gold contacts, while channel regions in figures are green regions. The clear picture of long chains of polymer fibre using brush coating is reported for the first time to the best of our knowledge.

The directional coating as compared with spin coating was also confirmed by UV-visible spectroscopy. When polymer chains were aligned, the pi–pi stacking distance was reduced; as a result, electrons require less energy to hop in between localized states as compared with randomly oriented polymer chains. So, polymer (PCDTPT in our case) will absorb more light, and less transmission will be observed in directionally oriented polymer. This attribute was confirmed by the deposition of PCDTPT through brush coating on a glass substrate of known transmission spectra and observed transmission spectra using UV-visible spectroscopy. Figure 2*c*,*d* represents the transmission spectra of brush- and spin-coated devices. Figure 2*c* clearly shows that transmission is less in parallel alignment with respect to parallel polarized light compared with perpendicular polarized light, and the negligible difference was seen in figure 2*d* for the spin-coated device. In brush-coated device, the transmission intensity of perpendicular polarized light should be greater than or equal to spin-coated device transmission intensity, but from figure 2*d*, transmission intensity was less as compared with spin-coated device, and transmission spectra followed the same pattern as in parallel polarized transmission spectra. This can be explained as follows: during the de-wetting process after deposition through brush coating some polymer chains were released from brush fibre stress and oriented in other directions, but still, their pi–pi stacking distance was reduced as compared with spin-coated device. This claim is also justified by electrical results.

**Figure 3.**
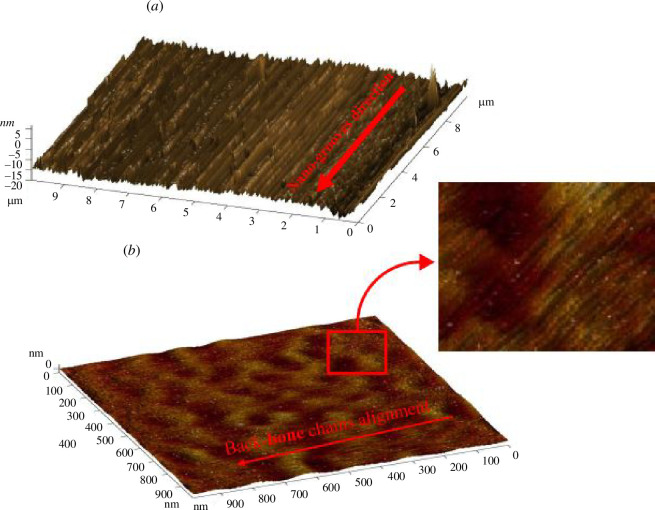
(*a*) AFM image of nano-grooved substrate prior to organic compound deposition. Root mean square height of nanogrooves are approximately 5 nm. (*b*) AFM image after PCDTPT deposition using brush coating. Long chain alignment can be seen from image along the direction of nanogrooves.

For in-depth learning and understanding of the nano-grooved configuration of the silicon substrate, an AFM analysis of the substrate was carried out. Figure 3*a* shows the AFM image of nano-grooved substrate prior to deposition. The width of nanogrooves is 100–200 nm and the root mean square height of grooves is approximately 5 nm. Figure 3*b* is the AFM image after PCDTPT deposition using brush coating. As the polymer was brush coated along the direction of nanogrooves, polymer chains were aligned along the nanogrooves. The inset clearly shows the alignment of polymer chains.

### 3.2. Electrical measurements

**Figure 4.**
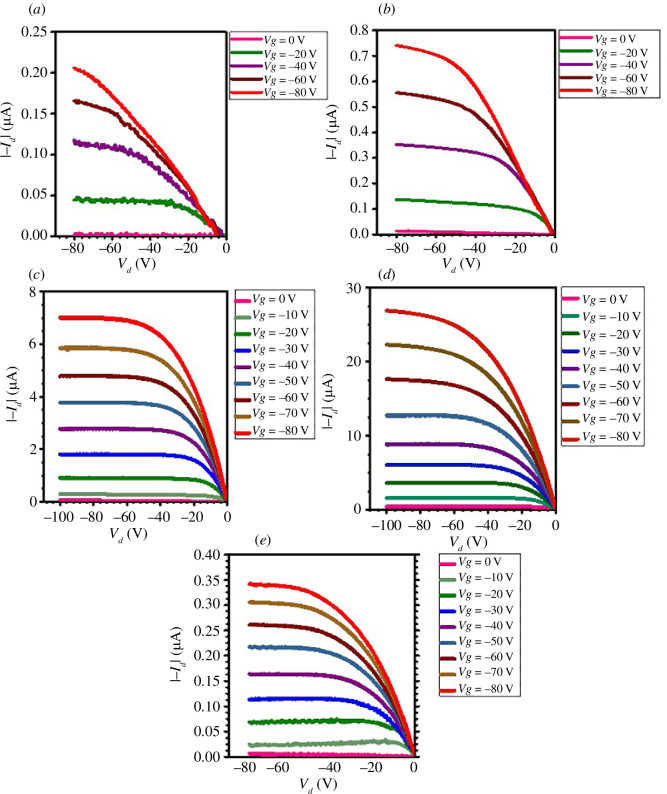
Output curves of (*a*) perpendicular dip-coated device, (*b*) parallel dip-coated device, (*c*) perpendicular brush-coated device, (*d*) parallel brush-coated device and (*e*) spin-coated device.

**Figure 5.**
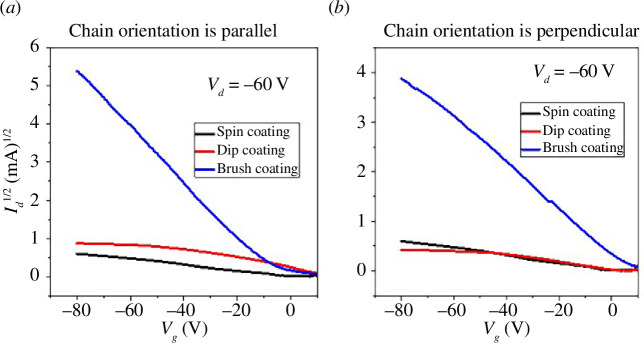
Combined transfer characteristics of parallel and perpendicular spin-, dip- and brush-coated devices, (*a*) when chain orientation is parallel, (*b*) when chain orientation is perpendicular.

Figure 4 shows representative output curves of all the devices. Figure 5 shows the transfer characteristics at saturation regime of best devices on each substrate with parallel and perpendicular channel orientation for spin-, dip- and brush-coated devices. Saturation mobility was extracted using the expression $\mu_{sat}=\frac{2L}{C_{i}W}{\left(\frac{d\sqrt{\left|I_{d}\right|}}{dV_{g}}\right)}^{2}$ and threshold voltage was extracted from linear fitting of square root current versus gate voltage graph. Here, *C*_ox_ is dielectric capacitance of PMMA/Si_3_N_4_ which is 10 nF cm^−2^. On each substrate there were four devices, two were perpendicular to the other two in terms of channel orientation. In dip coating device, there is an increase in current when channel orientation is parallel to the direction of polymer chains, as expected.

In brush-coated devices, the current is increased for both cases when channel orientation is parallel and perpendicular with respect to polymer chain alignment because of the spreading of some polymer chains in other directions, as mentioned above. However, at high gate voltages, for parallel channel orientation devices, the current is large as compared with perpendicular.

When channel orientation is parallel, one can see the increase in current. There is more right-side threshold voltage shift for dip-coated device as compared with brush-coated device. This random behaviour is expected in ambient conditions. For perpendicular channel orientation, dip-coated device behaved the same as spin-coated device, as expected ideally, because polymer chains are aligned in other directions as in spin-coated device. However, for brush-coated device in perpendicular channel orientation, there is an increase in current due to the spreading of polymer chains in other directions, and from figure 2, it can be seen some are aligned in channel length direction, which resulted in an increase in current.

**Figure 6.**
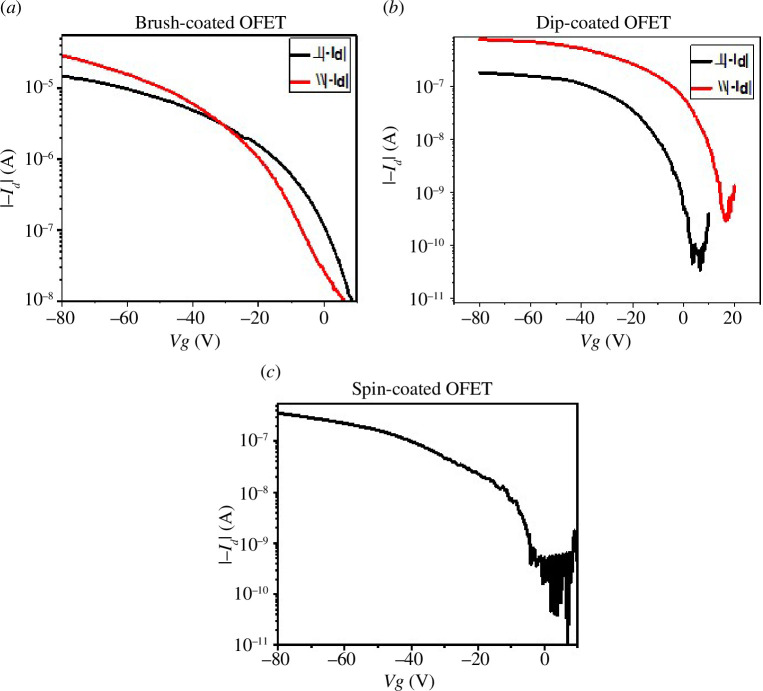
Transfer curves on log scale.

For spin coating devices, there is no difference in transfer characteristics for both parallel and perpendicular channel orientation due to the non-directional deposition process. But here we used the same nomenclature as we used for dip and brush coating devices. Transfer curves on log scale are also shown to represent the switching behaviour of the devices (figure 6). All the devices show reasonable current on–off ratio while brush-coated device for parallel alignment shows the best *I*_on_/*I*_off_ to be 2.9 × 10^5^. Table 1 shows current on–off ratio of all the devices.

**Table 1.** Current on–off ratio.

device	on/off ratio
brush-coated OFET //	2.9 × 10^5^
brush-coated OFET ⊥	1.4 × 10^5^
dip-coated OFET //	1.99 × 10^3^
dip-coated OFET ⊥	3.8 × 10^3^
spin-coated OFET	1.83 × 10^3^

**Figure 7.**
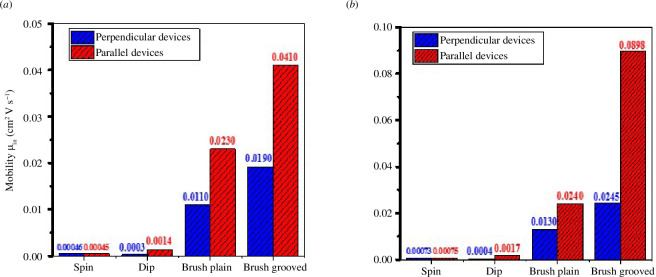
(*a,b*) Mobility comparison of the asymmetric devices (perpendicular and parallel to the grooves, respectively) for the linear and saturation regimes, respectively.

From figure 7*a*, for parallel channel orientation we can observe that there is two times increase in linear mobility for dip coating as compared with spin coating, and there is 55 times increase in mobility for brush coating as compared with spin coating device. When making a comparison between dip- and brush-coated devices for parallel channel orientations, there is 24 times increase in mobility. As mentioned earlier and can be seen in figure 7*a*, there is also 33 times increase in mobility for brush coated device when channel orientation is perpendicular compared to spin coated device. When making comparison for same brush coated devices for perpendicular and parallel orientation, there is 1.7 times increase in mobility for parallel orientation. A similar trend can be observed for saturation mobility in figure 7*b*. These findings suggest that brush coating is a potential and facile way to remarkably enhance the performance of OFETs.

## 4. Conclusions

The OFET devices were fabricated through spin, dip and brush coating techniques in ambient conditions by solution processing. For dip- and brush-coated devices, nanogrooves on substrates were grown using diamond lapping film. Long polymer chains were observed along the direction of nanogrooves when structural analysis was performed through optical microscope and AFM. From electrical measurements, we observed 90 times increase in linear mobility for brush-coated device when channel orientation was parallel to polymer chains compared with the control device based on spin coating. There was also 24 times increase in mobility for brush-coated device compared with the dip-coating device when channel orientation was parallel for both devices. We have performed a comprehensive study to give a comparison between different directional techniques such as dip coating, brush coating and nano-grooves patterning. From our findings, we conclude that the brush coating technique is a promising solution for low-cost large-area organic electronics in the ambient environment.

## Data Availability

The data are provided in the electronic supplementary material [32].
